# Newly Diagnosed Monostotic Paget’s Disease of Bone during Living Kidney Donor Candidate Evaluation

**DOI:** 10.3390/biomedicines11020401

**Published:** 2023-01-29

**Authors:** Diana Jędrzejuk, Paweł Poznański, Paweł Szewczyk, Oktawia Mazanowska, Marek Bolanowski, Magdalena Krajewska, Dorota Kamińska

**Affiliations:** 1 Department of Endocrinology, Diabetes and Isotope Therapy, Wrocław Medical University, 50-367 Wrocław, Poland; 2 Department of Nephrology and Transplantation Medicine, Wrocław Medical University, 50-556 Wrocław, Poland; 3 Department of General and Interventional Radiology and Neuroradiology, Jan Mikulicz–Radecki University Clinical Hospital in Wrocław, 50-556 Wrocław, Poland

**Keywords:** Paget’s disease of bone, bone scintigraphy, bone mineral density, trabecular bone score

## Abstract

The popularity of living-donor organ donation has increased recently as an alternative to deceased-organ donation due to the growing need for organs and a shortage of deceased-donor organs. This procedure requires an in-depth health assessment of candidates, who must be in excellent physical and mental health. We present a potential living-kidney donor withdrawn from donation due to a newly diagnosed Paget’s disease of bone (PDB). The patient underwent computed tomography (CT), magnetic resonance imaging (MRI), bone scintigraphy, and bone densitometry with trabecular bone score (TBS) assessment. The sole lumbar vertebra affected by PDB was investigated comprehensively, non-invasively, quantitatively, and qualitatively.

## 1. Introduction

Living-donor kidney transplantation (LDKT) is the treatment of choice for most patients with end-stage renal disease, offering optimum patient and graft survival and reduced time on the transplant waiting list. In such situations, donor welfare and care remain paramount. Medical and pre-operative evaluation and identification of high-risk donors are important. Assessment may reveal previously undiagnosed disease. Early detection of disease may benefit the donor and may also withdraw living-donor candidates from the transplant process. Living-donor candidates must meet health criteria included in local and international guidelines [1,2].

We present a case of a living-kidney donor candidate withdrawal due to monostotic Paget’s disease of bone (PDB) with vertebral localization. We have previously published a detailed discussion of the reasons for withdrawal [3].

## 2. Case Presentation

A living-donor kidney donation coordinator considered a 54-year-old male a potential donor for his daughter. His preliminary medical tests revealed no abnormalities. He was admitted to the Department of Nephrology and Transplantation Medicine for extended medical assessment. The patient self-declared good health and had no chronic diseases or complaints other than occasional back pain. 

The results of blood and urine tests, mandatory for eligibility to the living kidney-donor program (for example, blood cell count, tumor markers, and urine tests) [1,2], were normal. Concentrations of almost all serum biochemical markers were in the normal range; however, alkaline phosphatase (ALP) activity exceeded the reference range (maximal concentration was 199 U/L during six months of observation; normal range 40–129 U/L). The most important results in the context of calcium metabolism and the conversion of vitamin D into active forms are presented in Table 1; no other hormonal tests or markers of bone turnover were performed.

**Figure 1:**
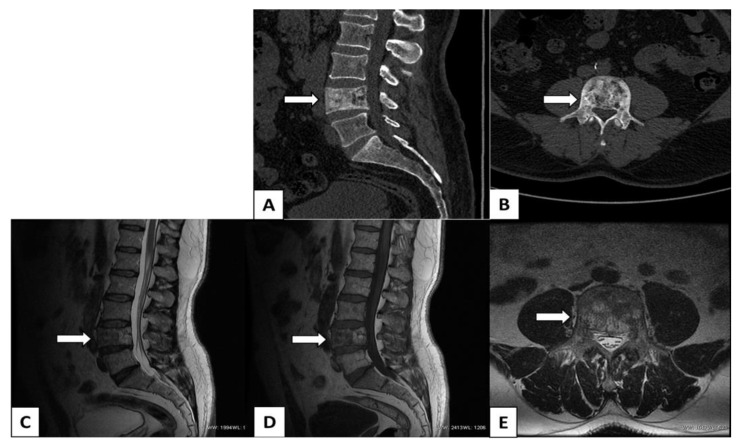
Computed tomography angiography (CTA) and magnetic resonance imaging (MRI): (**A**) Sagittal CT image, bone window, reveals mildly enlarged and “squared” L4 (arrow) vertebral body. The vertebral body is mostly sclerotic with cortical thickening and disorganized, coarsened vertical trabeculae. (**B**) Axial CT image, bone window, shows heterogenous sclerotic remodeling and end enlargement of the L4 vertebra with sclerotic cortical thickening. Changes also affect the vertebral pedicles and the neural arch of the vertebra. (**C**,**D**) Sagittal MRI T1-weighted image (T1WI) and T2WI of the lumbar spine reveal enlargement of the L4 vertebral body with mild vertebral canal stenosis. The L4 vertebral body is “squared” and shows loss of the anterior concave margin. Heterogeneous change of bone marrow signal of the affected vertebral body. In the anterior part, the low signal on T1WI and T2WI suggests sclerosis-fibrosis and late, blastic inactive phase of Paget’s disease of bone (PDB). In the posterior part, the high signal on T1WI and T2WI suggests fatty marrow of mixed (second) phase of PDB. (**E**) Axial MRI T2WI image shows an enlargement of the vertebral body and pedicles, heterogeneous bone marrow remodeling with thickened trabeculae, and patchy areas of high signal in the posterior part, suggesting fatty transformation.

Computed tomography angiography (CTA) and renal scintigraphy were performed for routine living-kidney donor qualification [4]. In renal scintigraphy, after 193 MBq I.V. ^99m^Technetium-diethylenetriamine-pentaacetic acid (^99m^Tc + DTPA) administration, both kidneys showed normal function (glomerular filtration rate 87.74 mL/min). In CTA, a hemangioma of the fourth lumbar vertebra (L4) with a chronic fracture was initially suspected (Figure 1A,B). This finding was followed up with magnetic resonance imaging (MRI), resulting in a diagnosis of Paget’s disease of bone (PDB) (Figure 1C–E) [5].

**Figure 2:**
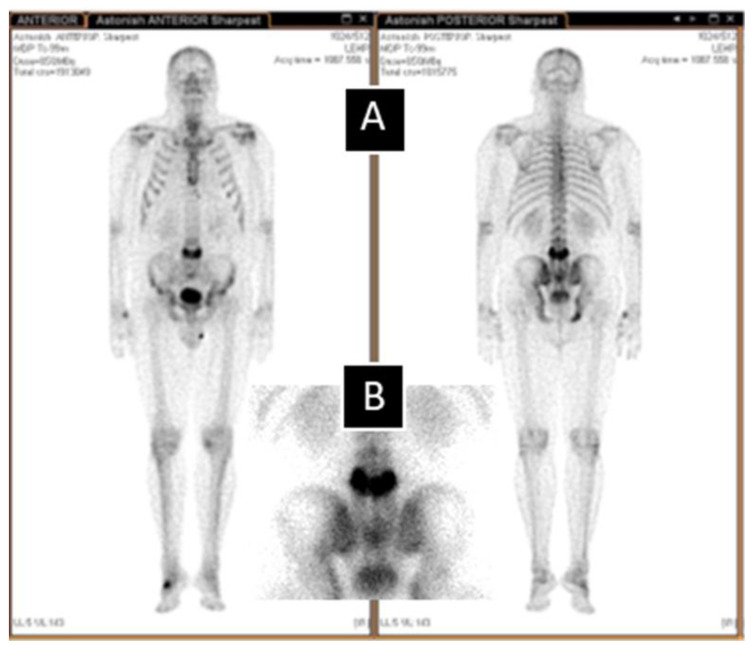
Bone scan images. (**A**) Whole body bone scan (anterior and posterior view) and (**B**) focused on the lumbar spine and pelvis.

To evaluate whether other bones were affected (monostotic or polyostotic PDB), a bone scan was performed 2 h post 740 MBq I.V. ^99m^Technetium-methylene diphosphonate (^99m^Tc + MDP) injection (Figure 2). The bone scan revealed an intensely increased radiopharmaceutical uptake throughout the fourth lumbar vertebra (L4), involving the body, posterior elements, and spinous process, referred to as the clover/heart/Mickey Mouse sign. In the case of patients with high ALP activity and without an oncological history, this symptom indicates a highly probable PDB diagnosis [6,7,8].

**Figure 3:**
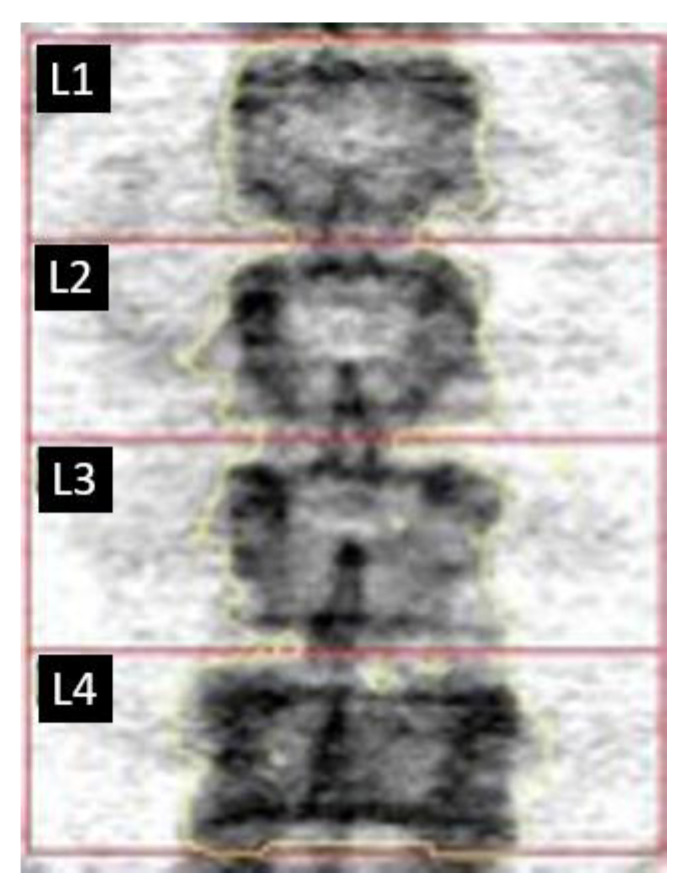
Densitometric image of the lumbar spine.

Further assessment of the pagetic vertebra involved a dual-energy X-ray absorptiometry (DXA), a gold standard in bone density measurement (Horizon A, Hologic, USA), followed by calculation of TBS (TBS iNsight v. 3.0.2.0) [9]. The lumbar spine densitometry allows assessing the bone density of each L1–L4 lumbar vertebra separately and together. The densitometric image of the lumbar spine is presented below (Figure 3) and should not be used to make any diagnosis. However, the fourth lumbar vertebra seems more calcified than other vertebrae; the color scale is the “negative” of the negative usually used in radiology; darker color = more calcified structure.

Low bone mineral density (BMD) was found in the lumbar spine (L1–L4; T-score −2.1). L4 had the highest BMD among the lumbar vertebrae examined, which corresponded to normal bone density. Lumbar vertebrae T-scores for L1–L3 indicated osteoporosis; each vertebra had a T score < −2.5 (Table 2A). L4 also presented the highest TBS compared to other lumbar vertebrae (Table 2B) [9,10].

**Figure 4:**
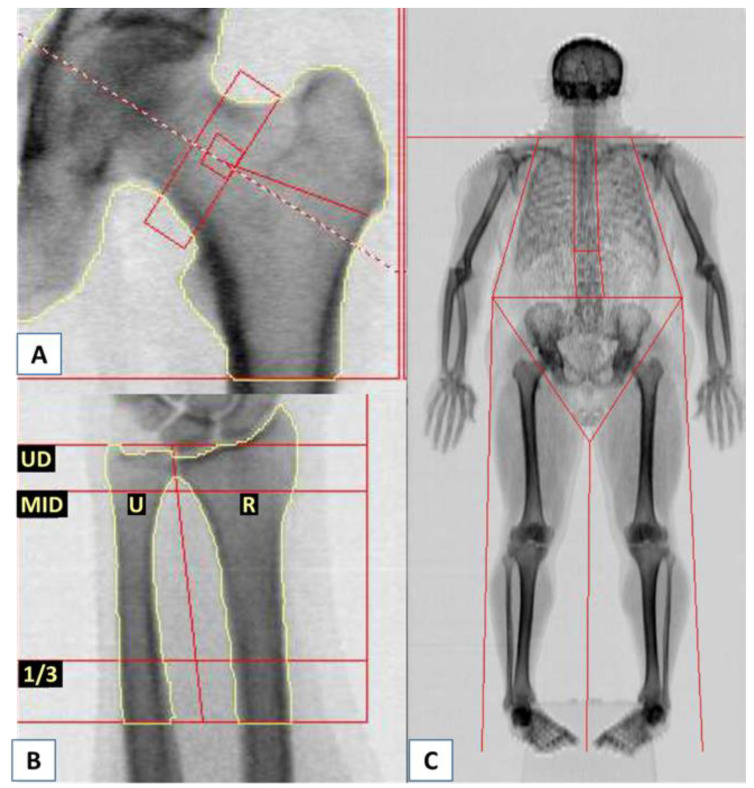
DXA scans of the left femoral neck (**A**), forearm (**B**), and total body (**C**). **Abbreviations:** 1/3, distal cortical part of 1/3 radius + ulna; MID, midshaft radius + ulna; R, radius; U, ulna; UD, ultradistal radius + ulna.

BMD was also measured in other locations: the femoral neck, non-dominant forearm, and total body (Figure 4). The BMD values were not below low bone mass, meaning osteoporosis could not be diagnosed in these locations (Table 2C). The non-dominant forearm showed low bone density: in the ultradistal and one-third distal sections of the forearm. Those values were similar to total body BMD. BMD was within normal ranges only in the femoral neck.

Considering all available patient information, we calculated the 10-year probability of fracture (%) using the Fracture Risk Assessment Tool, FRAX [11]. The major osteoporotic fracture (MOF) probability was 3.3% and for hip fracture was 0.1%. After adjustment for TBS, the probabilities were 6.3% and 0.2%, respectively.

According to the National Osteoporosis Foundation guidelines, patients with FRAX 10-year risk scores of ≥20% for MOF or ≥3% for hip fracture should be treated [12].

The above results, which led to PDB diagnosis and the analysis of the benefits and disadvantages of kidney donation as a living donor, resulted in the patient’s withdrawal from the living kidney donor program [3]. The patient was referred to the Rheumatology Department for further evaluation and treatment, where he qualified for risedronate use. In follow-up examinations after one year, ALP activity decreased from 199 to 69 U/L (normal range 40–129 U/L), the intensity of radiotracer uptake on the bone scan decreased, and BMD of the lumbar spine increased insignificantly. However, the MRI image did not change significantly. The patient is still under periodic observation at the Rheumatology Department.

## 3. Discussion

PDB is a chronic, metabolic bone disease. The information on the prevalence of PDB was published in 2013, and its prevalence varied in the different countries studied; the highest prevalence rate was reported in the UK (>5%), then in western and southern Europe, but uncommon or rare in Scandinavia, on the Indian subcontinent, in Southeast Asia, and Japan (<0.0003%) [13,14]. In Poland, PDB is rarely diagnosed, and the epidemiological data is scarce but suggestive of a declining incidence [15]. Our patient’s case is extremely rare, as he was diagnosed with PDB due to participation in the living kidney donor program, requiring numerous tests. This information led to considerations about the possible future of the patient, who would probably be exposed to painkillers (bone pain) and bisphosphonate (bone pain and bone turnover reduction effect), which could damage his one remaining kidney [3]. Ultimately, the patient was withdrawn from the living-donor kidney program.

However, despite the relatively small number of people with PDB, the annual number of publications on PDB fluctuates rather than decreases. For example, recently, there have been publications from Asia [16,17,18] and more papers about the genetic aspects of PDB [16,19,20].

The etiology of PDB remains uncertain; genetic and environmental factors (paramyxoviruses) are suggested. A family history is present in at least 15% of cases, with the risk of developing the disease by a relative of a PBD patient being 7–10 times greater than in the general population [13]. Still, the genetic cause remains unknown in up to 50% of familial patients [19]. In the etiology of PDB, attention has recently been paid to the role of the RANKL/OPG/RANK pathway [16,20].

In PDB, abnormalities such as unusual bone growth presents in several ways. PDB involves excess osteoclastic activity followed by a compensatory increase in osteoblastic activity, leading to disorganized bone formation. The primary disorder is higher bone turnover [21,22]. Therefore, the most frequent therapy involves bisphosphonates, which interfere with osteoclast function and decrease bone turnover [23,24].

Nowadays, diagnosis of PDB is usually a secondary finding on an abnormal X-ray, CT, MRI, or hybrid imaging (positron emission tomography and CT; PET/CT) [5,25,26,27,28,29,30,31] and/or elevated alkaline phosphatase activity of an unknown etiology, as in our patient [21,24]. The characteristics listed above that lead to the diagnosis are related to earlier and wider access to medical examinations. Using various imaging methods facilitates making the correct diagnosis of PDB and avoids unnecessary biopsy [32]. Clinical features include bone pain, deformity, pathologic fracture, secondary osteoarthritis, and deafness. Significantly less common are spinal stenosis, nerve compression syndromes, hypercalcemia, hydrocephalus, paraplegia, cardiac failure, and osteosarcoma [21].

The most common PDB involvement sites are the pelvis, lumbar spine, and femur, reported in more than 75% of cases, with the polyostotic disease being more common than the monostotic [13], but the axial skeleton is usually involved. It is assumed that about 75% of patients are asymptomatic [22], although some studies indicate that pain in the affected site is the most common symptom [25,26,27], along with deformity and fracture [22,25]. PDB occurs more often in men than women and is uncommon in people under 50. However, PDB is the second most common bone disorder in elderly individuals and affects 7% of men and 6% of women over the age of 85 in the UK [13,22].

Diagnosis of PDB based on radiological tests (X-ray, MRI, and CT) has been extensively described [33,34,35,36].

Bone scintigraphy is the most sensitive and economical way of detecting PDB, determining the monostotic or polyostotic disease type, or differentiating the etiology of low back pain in uncertain diagnosis. Bone scans are useful not only to evaluate the entire skeleton for PDB but also to screen for complications associated with PDB, such as fractures and malignant transformations, and to monitor the response to therapy. Increased radiotracer uptake is found in all PDB phases because the osteoblastic activity is present from the early stage [37]. Therefore, a bone scan with 99mTc-labeled phosphonate derivatives is very sensitive imaging and rather specific for PDB; a trained clinical nuclear medicine specialist should not have any problem differentiating it from other bone diseases. However, the Mickey Mouse sign is not unique to PDB alone; this sign and other signs typical for PDB detected on a bone scan can be recognized as metastases, mimic osseous metastases, or coexist with metastases [32,38,39,40]. Sometimes the differential diagnosis can also be metabolic bone disease or even fibrous dysplasia [41]. The radiotracer uptake in PDB patients is intense and well-demarcated. In long bones, pagetic lesions appear at the articular margin, progressing along the shaft and producing a sharp V-shaped advancing edge such as a flame (flame sign); it is clearly visible on X-rays and bone scans [41]. In contrast, metastatic disease may present with asymmetrical, irregular, heterogeneous, and spotty radiopharmaceutical uptake [42].

Although the number of X-ray, CT, and MRI examinations performed is much greater than bone scans or PET/CT, shortly, there will be more new, accidental PDB diagnoses during radionuclide examinations than in typical, classical radiology. This is related to the increasing use of nuclear medicine scans, which involve examining the whole body in a single test.

Bone densitometry allows assessing the bone density of each L1–L4 lumbar vertebra separately and together. What is even more interesting, all bone densitometers have programs for evaluating only the lumbar spine (the BMD of the thoracic or cervical spine cannot be measured). There are a few articles about bone mineral density (BMD) or its derivates in pagetic patients [43,44,45]. However, there are no papers where both BMD and TBS were measured in a vertebra (or other localization that can be measured using bone densitometry) affected by PDB. Pagetic bone is known to have high or extremely high BMD because the bone is larger and the “density” measured by densitometry is only “planar” [43,45]. In our patient, BMD results in all examined locations (lumbar spine, femoral neck, forearm, and total body) did not meet the WHO criteria for the diagnosis of normal bone mass (T-score > −1.0) (see Table 2A). This may be due to the diagnosis of the disease at an early stage, relatively weak osteoblastic activity, and a small fragment of affected bone (L4 only). In our study, the affected L4’s BMD also did not meet the conditions of “elevated bone mass,” proposed by J. Paccou et al. as Z-score ≥ +4.0 [46]. The result of the L4’s Z-score in our patient was −0.4 and was also the highest Z-score of all examined vertebrae.

The use of densitometry in bone density assessment is almost universal, but the study of bone texture with TBS is not widely available. TBS is derived from the texture of the DXA image and is shown to be related to bone microarchitecture and fracture. This data provides information independent of BMD. It complements the data obtained from DXA and clinical examination. In our patient, TBS of the affected vertebra was higher than the rest of the vertebrae examined (clinical interpretation “partially degraded” for L4 and “degraded” for the other vertebrae) (see Table 2B). Higher TBS indicates that bone texture is not as degraded as, for example, in low bone mass, osteoporosis, and hypercortisolism [9], probably also because the changes are unrelated to bone calcium content and, above all, bone mineral content (BMC) in L4 is also the highest (Table 2A). Pande et al. showed that the quality parameters derived from quantitative ultrasound (QUS) assessment, complementary bone density testing to measurement by DXA, do not indicate bone thinning; the speed of sound (SOS) (m/s) was lower than in normal, non-pagetic bone [44]. Thus, non-invasive methods showed higher BMD and higher bone quality in PDB-affected bones.

Our patient was referred to the Rheumatology Department to be treated for PDB of L4 and accompanying “low bone mass” in densitometric examinations of other localizations. However, there were no indications for treatment due to fracture risk (low MOF and hip fracture probability). The most common treatment of PDB is an intravenous infusion of bisphosphonates, recently zoledronic acid [3,21]. In Poland, zoledronic acid is registered only for treating neoplastic hypercalcemia and preventing fractures/bone complications in oncology patients. For that reason, our patient was treated with risedronate: one tablet every two weeks. As expected, after one year of treatment, the serum activity of ALP decreased (69 U/L), the intensity of radiopharmaceutical uptake in L4 decreased and BMD at the lumbar spine increased insignificantly.

Imaging has a crucial role in the diagnosis and follow-up of Paget’s disease. The imaging modalities are complementary, e.g., CT, which provides details of bony architecture and functional imaging (bone scan), which is useful to demonstrate the bone turnover activity of the whole skeleton. Therefore, hybrid imaging, such as PET/CT and single-photon emission computed tomography with CT (SPECT/CT), plays a vital role in diagnosis and monitoring. Furthermore, novel nuclear medicine technologies (including new cadmium-zinc-telluride detectors in SPECT cameras) facilitate a whole-body study in three dimensions, not only as a CT but also as a nuclear scan in a very short time (thus far, full body scanners giving AP and PA planar information are usually used) [47].

## 4. Conclusions

To our knowledge, this is the first report of a vertebra affected by PDB diagnosed, which was confirmed using CT, MRI, and a bone scan, assessed using DXA (the results of BMD and TBS). In this case, BMD and TBS measurements in the lumbar vertebra accurately show how the density and texture of healthy and pagetic vertebrae differ.

Increased BMD, TBS of L4, and high ALP activity with typical signs on CT, MRI, and bone scans confirmed PDB diagnosis, and the patient avoided bone biopsy. However, CT scans alone can reveal highly vascular lesions with lysis and sclerosis within the same structure. As a result, hemangioma with a chronic fracture can be initially diagnosed, as in this case. Therefore, atypical images or radiological/scintigraphic findings should always be supplemented with other imaging (morphological or radionuclide) and correlated with the clinical status and additional results, e.g., blood serum.

## Figures and Tables

**Table 1:** Results of selected blood serum tests.

	Parameter/Reference Range	Value
Hormones	25 (OH) Vitamin D [30–50 ng/mL]	39.8
	PTH [15–68.3 pg/mL]	28.4
	TSH [0.35–4.94 mIU/mL]	1.288
Kidneys	Creatinine [0.8–1.3 mg/dL]	0.9
	eGFR [>60 mL/min/1.73 m^2^]	89
	Urea [17–43 mg/dL]	40
Liver	ALT [0–45 U/L]	31
	AST [0–35 U/L]	27
	Total bilirubin [0.2–1.2 mg/dL]	0.5
	Total protein [6.6–8.3 g/dL]	7
Macroelements	Total calcium [8.8–10.6 mg/dL]	9.8
	Magnesium [1.8–2.6 mg/dL]	2.0
	Inorganic phosphate [2.5–4.5 mg/dL]	4.1
Other	Ultrasensitive CRP [0–5 mg/L]	0.56
	Free PSA [>0.25 ng/mL]	0.3
	PSA [<4 ng/mL]	0.631
	Transferrin [20–250 g/L]	122.8

Abbreviations: ALT, alanine aminotransferase; AST, aspartate aminotransferase; CRP, C-reactive protein; eGFR, estimated glomerular filtration rate; PSA, prostate-specific antigen; PTH, parathyroid hormone; TSH, thyroid-stimulating hormone.

**Table 2:** Lumbar spine bone density (DXA), TBS results and BMD results.

(A). Lumbar Spine BMD Results Assessed by DXA.							
**Region**	**Area cm^2^**	**BMC g**	**BMD g/cm^2^**	**T-Score**	**PR%**	**Z-Score**	**AM %**
L1	15.42	12.20	0.791	−2.6	74	−2.1	77
L2	16.95	13.26	0.782	−2.8	71	−2.4	75
L3	18.40	15.09	0.820	−2.6	74	−2.1	78
L4	22.48	22.16	0.986	−0.9	90	−0.4	95
L1–L4	73.26	62.70	0.856	−2.1	78	−1.7	82
**(B). TBS Results in Relation to Lumbar Spine BMD.**							
**Region**	**TBS**	**TBS T-Score**	**TBS Z-Score**	**BMD g/cm^2^**			
L1	1.067	-	-	0.791			
L2	1.186	-	-	0.782			
L3	1.189	-	-	0.820			
L4	1.316	-	-	0.986			
L1–L4	1.190	−2.4	−1.4	0.856			
L1–L3	1.147	−2.6	−1.6	0.798			
L1–L2	1.127	−2.3	−1.4	0.786			
L2–L3	1.187	−2.7	−1.7	0.802			
L2–L4	1.230	−2.5	−1.4	0.873			
L3–L4	1.252	−2.5	−1.5	0.911			
**(C). BMD Results Assessed by DXA at other Sites (Femoral Neck, Forearm, and Total Body).**							
**Region**	**Area cm^2^**	**BMC g**	**BMD g/cm^2^**	**T-Score**	**PR%**	**Z-Score**	**AM %**
Femoral neck	6.33	5.87	0.927	+0.0	100	+0.8	114
Ultradistal part of non-dominant (left) forearm; radius and ulna	7.57	3.79	0.500	−0.2	98	+0.5	106
1/3 Distal part of non-dominant (left) forearm; radius and ulna	6.28	4.79	0.762	−1.1	92	−0.5	96
Total body	2330.89	2502.36	1.074	−1.3	90	−1.0	91

According to the World Health Organization (WHO): T-scores of −1.0 or above indicate normal bone density; T-scores between −1.0 and −2.49 indicate low bone density (previously called osteopenia); T-scores of −2.5 or below indicate osteoporosis. BMD difference between L4 and other lumbar vertebrae is 17−21%. TBS of L1–L4 is related to the degraded bone texture (<1.200). TBS of L4 was the highest of all lumbar vertebrae, typical of partially degraded bone texture (1.200 < TBS < 1.350). The difference in TBS values between L4 and other lumbar vertebrae is 10–19%. AM, age-matched; BMC, bone mineral content; BMD, bone mineral density; DXA, dual-energy X-ray absorptiometry; PR, peak reference; TBS, trabecular bone score; T-score, the number of standard deviations above or below the mean reference value for young healthy adults; Z-score, the number of standard deviations above or below the mean reference value for age and gender.

## Data Availability

All data and materials were included in this paper.

## References

[1] Andrews P.A., Burnapp L. (2018). British Transplantation Society/Renal Association UK Guidelines for Living Donor Kidney Transplantation 2018: Summary of Updated Guidance. Transplantation.

[2] Lentine K.L., Kasiske B.L., Levey A.S., Adams P.L., Alberú J., Bakr M.A., Gallon L., Garvey C.A., Guleria S., Li P.K. (2017). KDIGO Clinical Practice Guideline on the Evaluation and Care of Living Kidney Donors. Transplantation.

[3] Poznański P., Lepiesza A., Jędrzejuk D., Mazanowska O., Bolanowski M., Krajewska M., Kamińska D. (2022). Is a Patient with Paget’s Disease of Bone Suitable for Living Kidney Donation?—Decision-Making in Lack of Clinical Evidence. J. Clin. Med..

[4] Rasała J., Szczot M., Kościelska-Kasprzak K., Szczurowska A., Poznański P., Mazanowska O., Małkiewicz B., Dębiński P., Krajewska M., Kamińska D. (2020). Computed Tomogra-phy Parameters and Estimated Glomerular Filtration Rate Formulas for Peridonation Living Kidney Donor Assessment. Transplant. Proc..

[5] Morales H. (2015). MR Imaging Findings of Paget’s Disease of the Spine. Clin. Neuroradiol..

[6] Rotés-Sala D., Monfort J., Solano A., Miralles E., Vila J., Carbonell J. (2004). The clover and heart signs in vertebral scin-ti-graphic images are highly specific of Paget’s disease of bone. Bone.

[7] Van Heerden B.B. (1994). Mickey Mouse sign in Paget’s disease. J. Nucl. Med..

[8] Kim C.K., Estrada W.N., Lorberboym M., Pandit N., Religioso D.G., Alavi A. (1997). The “mouse face” appearance of the verte-brae in Paget’s disease. Clin. Nucl. Med..

[9] Gonera-Furman A., Bolanowski M., Jędrzejuk D. (2022). Osteosarcopenia-The Role of Dual-Energy X-ray Absorptiometry (DXA) in Diagnostics. J. Clin. Med..

[10] Koo M., Chuang T.L., Wang Y.F. (2021). Research trends in trabecular bone score: A bibliometric review from 2008 to 2019. Tzu Chi Med. J..

[11] Chakhtoura M., Dagher H., Sharara S., Ajjour S., Chamoun N., Cauley J., Mahfoud Z., Boudreau R., El Hajj Fuleihan G. (2021). Systematic review of major osteoporotic fracture to hip fracture incidence rate ratios worldwide: Implications for Fracture Risk Assessment Tool (FRAX)-derived es-timates. J. Bone Miner Res..

[12] Siris E.S., Baim S., Nattiv A. (2010). Primary care use of FRAX: Absolute fracture risk assessment in postmenopausal women and older men. Postgrad Med..

[13] Gruener G., Camacho P. (2014). Paget’s disease of bone. Handb. Clin. Neurol..

[14] Corral-Gudino L., Borao-Cengotita-Bengoa M., Del Pino-Montes J., Ralston S. (2013). Epidemiology of Paget’s disease of bone: A systematic review and meta-analysis of secular changes. Bone.

[15] Kanecki K., Nitsch-Osuch A., Goryński P., Bogdan M., Tarka P., Tyszko P.Z. (2018). Paget disease of bone among hospitalized patients in Poland. Ann. Agric. Environ. Med..

[16] Xue J.Y., Ikegawa S., Guo L. (2021). Genetic disorders associated with the RANKL/OPG/RANK pathway. J. Bone Miner. Metab..

[17] Asirvatham A.R., Kannan S., Mahadevan S., Balachandran K., Sampathkumar G., Sadacharan D., Balasubramanian S.K. (2020). Is Paget Disease of Bone more Common in South India? Clinical Characteristics, Therapeutic Outcome and follow-up of 66 Patients from Tamil Nadu. Indian J. Endocrinol. Metab..

[18] Saito-Hakoda A., Kikuchi A., Takahashi T., Yokoyama Y., Himori N., Adachi M., Ikeda R., Nomura Y., Takayama J., Kawashima J. (2022). Familial Paget’s disease of bone with ocular manifestations and a novel TNFRSF11A duplication variant (72dup27). J. Bone Miner. Metab..

[19] Merlotti D., Materozzi M., Bianciardi S., Guarnieri V., Rendina D., Volterrani L. (2020). Mutation of PFN1 Gene in an Early On-set, Polyostotic Paget-like Disease. J. Clin. Endocrinol. Metab..

[20] Gennari L., Rendina D., Merlotti D., Cavati G., Mingiano C., Cosso R., Materozzi M., Pirrotta F., Abate V., Calabrese M. (2022). Update on the pathogenesis and genetics of Paget’s disease of bone. Front. Cell Dev. Biol..

[21] Ralston S.H., Corral-Gudino L., Cooper C., Francis R.M., Fraser W.D., Gennari L., Guañabens N., Javaid M.K., Layfield R., O’Neill T.W. (2019). Diagnosis and Management of Paget’s Disease of Bone in Adults: A Clinical Guideline. J. Bone Miner. Res..

[22] Bouchette P., Boktor S.W. (2022). Paget Disease. [Updated 2021 Jul 13]. StatPearls [Internet].

[23] Corral-Gudino L., Tan A.J.H., Del Pino-Montes J., Ralston S.H. (2017). Bisphosphonates for Paget’s disease of bone in adults (Review). Cochrane Database Syst. Rev..

[24] Choi Y.J., Sohn Y.B., Chung Y.S. (2022). Updates on Paget’s Disease of Bone. Endocrinol. Metab..

[25] Appelman-Dijkstra N.M., Papapoulos S.E. (2018). Paget’s disease of bone. Best Pract. Res. Clin. Endocrinol. Metab..

[26] Konidala J., Raj R. (2022). Monostotic humeral Paget’s disease. BMJ Case Rep..

[27] Feki A., Sellami I., Gassara Z., Ben Djemaa S., Ezzeddine M., Kallel M.H., Fourati H., Akrout R., Baklouti S. (2022). Spinal Paget’s disease with bilevel cord compression and ischemic non-compressive myelopathy treated with zoledronic acid. Clin. Case Rep..

[28] Cook G.J., Blake G.M., Marsden P.K., Cronin B., Fogelman I. (2002). Quantification of skeletal kinetic indices in Paget’s disease using dynamic 18F-fluoride positron emission tomography. J. Bone Miner. Res..

[29] Installe J., Nzeusseu A., Bol A., Depresseux G., Devogelaer J.P., Lonneux M. (2005). 18F-Fluoride PET for Monitoring Therapeu-tic Response in Paget’s Disease of Bone. J. Nucl. Med..

[30] Artigas C., Alexiou J., Garcia C., Wimana Z., Otte F.X., Gil T., Van Velthoven R., Flamen P. (2016). Paget bone disease demonstrated on 68Ga-PSMA ligand PET/CT. Eur. J. Nucl. Med. Mol. Imaging.

[31] Dondi F., Albano D., Treglia G., Bertagna F. (2022). Paget Disease as Common Pitfall on PET with Different Radiopharmaceuti-cals in Oncology: Not All That Glitters Is Gold!. J. Clin. Med..

[32] Kesim S., Turoğlu H.T., Özgüven S., Öneş T., Erdil T.Y. (2020). Mickey Mouse Sign on Bone Scan in the Monostotic Form of Paget’s Disease Mimicking Osseous Metastasis. Mol. Imaging Radionucl. Ther..

[33] Whitehouse R.W. (2002). Paget’s disease of bone. Semin Musculoskelet Radiol..

[34] Winn N., Lalam R., Cassar-Pullicino V. (2017). Imaging of Paget’s disease of bone. Wien. Med. Wochenschr..

[35] Whitten C.R., Saifuddin A. (2003). MRI of Paget’s disease of bone. Clin. Radiol..

[36] Lombardi A.F., Aihara A.Y., Fernandes A.D.R.C., Cardoso F.N. (2022). Imaging of Paget’s Disease of Bone. Radiol. Clin. N. Am..

[37] Love C., Din A.S., Tomas M.B., Kalapparambath T.P., Palestro C.J. (2003). Radionuclide Bone Imaging: An Illustrative Review. Radiographics.

[38] Fukushi K., Koie T., Yamamoto H., Okamoto A., Imai A., Hatakeyama S., Yoneyama T., Hashimoto Y., Ohyama C. (2013). [Paget’s disease mimicking metastatic prostate cancer on bone scan image: A case report]. Hinyokika Kiyo..

[39] Khan F.M., Chen B., Takalkar A.M. (2022). Mickey Mouse Sign on Bone Scan: This Mouseketeer Rebels Against Paget Disease. Clin. Nucl. Med..

[40] Aiba H., Nakazato T., Matsuo H., Kimura H., Saito S., Sakai T., Murakami H., Kawai J., Kawasaki S., Imamura Y. (2022). Bone Metastases from Gastric Cancer Resembling Paget’s Disease: A Case Report. J. Clin. Med..

[41] Kumar A.A., Kumar P., Prakash M., Tewari V., Sahni H., Dash A. (2013). Paget’s disease diagnosed on bone scintigraphy: Case report and literature review. Indian J. Nucl. Med..

[42] Shetty S., Shetty S., Prabhu A.J., Kapoor N., Hepzibah J., Paul T.V. (2016). An unusual presentation of metastatic bone disease in a subject with Paget’s disease of bone. J. Family Med. Prim. Care.

[43] Cherian R.A., Haddaway M.J., Davie M.W.J., McCall I.W., Cassar-Pullicino V.N. (2000). Effect of Paget’s disease of bone on areal lumbar spine bone mineral density measured by DXA, and density of cortical and trabecular bone measured by quantita-tive CT. Br. J. Radiol..

[44] Pande K.C., Bernard J., McCloskey E.V., de Takats D., Kanis J.A. (2000). Ultrasound velocity and dual-energy X-ray absorptiom-etry in normal and pagetic bone. Bone.

[45] Polyzos S.A., Anastasilakis A.D., Litsas I., Sapranidis M., Efstathiadou Z., Kita M., Arsos G., Moralidis E., Zafeiriadou E., Papatheodorou A. (2010). Dual-energy X-ray absorptiometry and quantitative ultrasound in patients with Paget’s dis-ease of bone before and after treatment with zoledronic acid: Association with serum bone markers and Dickkopf-1. J. Clin. Densitom..

[46] Paccou J., Javier R.M., Henry-Desailly I., Ternynck C., Nottez A., Legroux-Gérot I., Robin F., Fardellone P., Lespessailles E., Roux C. (2021). The French multicentre elevated bone mass study: Prevalence and causes. Osteoporos Int..

[47] Yamane T., Takahashi M., Matsusaka Y., Fukushima K., Seto A., Kuji I., Matsunari I. (2021). Satisfied quantitative value can be acquired by short-time bone SPECT/CT using a whole-body cadmium-zinc-telluride gamma camera. Sci Rep..

